# Testing of Materials and Elements in Civil Engineering

**DOI:** 10.3390/ma14123412

**Published:** 2021-06-20

**Authors:** Krzysztof Schabowicz

**Affiliations:** Faculty of Civil Engineering, Wrocław University of Science and Technology, Wybrzeże Wyspiańskiego 27, 50-370 Wrocław, Poland; krzysztof.schabowicz@pwr.edu.pl

**Keywords:** testing, diagnostic, building materials, elements, civil engineering

## Abstract

This issue is proposed and organized as a means to present recent developments in the field of testing of materials in civil engineering. For this reason, the articles highlighted in this issue should relate to different aspects of testing of different materials in civil engineering, from building materials and elements to building structures. The current trend in the development of materials testing in civil engineering is mainly concerned with the detection of flaws and defects in elements and structures using destructive, semi-destructive, and nondestructive testing. The trend, as in medicine, is toward designing test equipment that allows one to obtain a picture of the inside of the tested element and materials. Very interesting results with significance for building practices of testing of materials and elements in civil engineering were obtained.

## 1. Introduction

The field of testing of materials in civil engineering is very wide, and is interesting from an engineering and scientific point of view [1,2,3]. This issue is proposed and organized as a means to present recent developments in the field of testing of materials in civil engineering. For this reason, the articles highlighted in this issue should relate to different aspects of testing of different materials in civil engineering, from building materials and elements to building structures [4,5,6]. The current trend in the development of materials testing in civil engineering is mainly concerned with the detection of flaws and defects in elements and structures using destructive, semi-destructive, and nondestructive testing. 

This issue mainly focuses on different novel testing approaches, the development of single and hybrid measurement techniques, and advanced signal analysis. The topics of interest include but are not limited to the testing of materials and elements in civil engineering, testing of structures made of novel materials [7,8,9], condition assessment of civil materials and elements, detecting defects invisible on the surface, damage detection and damage imaging, diagnostics of cultural heritage monuments, structural health monitoring systems, modeling and numerical analyses, nondestructive testing methods, and advanced signal processing for nondestructive testing [10,11].

## 2. Description of the Articles Presented in the Issue 

Grouted rock bolts represent one of the most used elements for rock mass stabilization, as analyzed by [12], and reinforcement and the grouting quality have a crucial role in the load transfer mechanism. At the same time, the grouting quality, as well as the grouting procedures, are the least controlled in practice. This paper deals with the non-destructive investigation of grouting percentage through an analysis of the rock bolt’s natural frequencies after applying an artificial longitudinal impulse to its head by using a soft-steel hammer as a generator. A series of laboratory models, with different positions and percentages of the grouted section, simulating grouting defects, were tested. A comprehensive statistical analysis was conducted and a high correlation between the grouting percentage and the first three natural frequencies of rock bolt models has been established. After validation of FEM numerical models based on experimentally obtained values, a further analysis includes consideration of grout stiffness variation and its impact on the natural frequencies of rock bolts [12].

In their paper [13], in order to create and make available design guidelines, recommendations for energy audits, data for analysis and simulation of the condition of masonry walls susceptible to biological corrosion, deterioration of comfort parameters in rooms, or deterioration of thermal resistance, were given. The paper analyzes various types of masonry wall structures occurring in and commonly used in historical buildings over the last 200 years. The summary is a list of results of particular types of masonry walls and their mutual comparison. On this basis, a procedure path has been proposed which is useful for monitoring heat loss, monitoring the moisture content of building partitions, and improving the hygrothermal comfort of rooms. The durability of such constructions has also been estimated, and the impact on the condition of the buildings that have been preserved and are still in use today was assessed [13].

In [14], the laboratory testing of the construction materials and elements is a subset of activities inherent in sustainable building materials engineering. Two questions arise regarding the test methods used: the relation between test results and material behavior in actual conditions on the one hand, and the variability of results related to uncertainty on the other. The paper presents the analysis of the results and uncertainties of the two simple independent test examples (bond strength and tensile strength) in order to demonstrate discrepancies related to the ambiguous methods of estimating uncertainty, and the consequences of using test methods when method suitability for conformity assessment has not been properly verified. These examples are the basis for opening a discussion on the test methods development direction which makes it possible to consider them as “sustainable”. The paper addresses the negative impact of the lack of complete test models, taking into account proceeding with the uncertainty regarding erroneous assessment risks. Adverse effects can be minimized by creating test methods appropriate for the test’s purpose (e.g., initial or routine tests) and handling uncontrolled uncertainty components. Sustainable test methods should ensure a balance between widely defined tests and evaluation costs and the material’s or building’s safety, reliability, and stability [14].

The article by [15] presents the possibilities of using foamed asphalt in the recycling process to produce the base layer of road pavement constructions, in Polish conditions. Foamed asphalt was combined with reclaimed asphalt pavement (RAP) and hydraulic binder (cement). Foamed asphalt mixtures with cement (FAC) were made, based on these ingredients. To reduce stiffness and cracking in the base layer, foamed asphalt (FA) was additionally used in the analyzed mixes containing cement. The laboratory analyses allowed estimating the stiffness and fatigue durability of the conglomerate. In the experimental section, measurements of deflections are made, modules of pavement layers are calculated, and their fatigue durability is determined. As a result of the research, new fatigue criteria for FAC mixtures and the correlation factors of stiffness modulus and fatigue durability in situ with the results of laboratory tests were developed. It is anticipated that FAC recycling technology will provide durable and safe road pavements [15].

The article by [16] presents experimental tests of a new type of composite bar that has been used as shear reinforcement for concrete beams. In the case of shearing concrete beams reinforced with steel stirrups, according to the theory of plasticity, the plastic deformation of stirrups, and stress redistribution in stirrups cut by a diagonal crack, are permitted. Tensile composite reinforcement is characterized by linear-elastic behavior throughout the entire strength range. The most popular type of shear reinforcement is closed-frame stirrups, and this type of fiber-reinforced polymer (FRP) shear reinforcement was the subject of research by other authors. In the case of FRP stirrups, rupture occurs rapidly, without the shear reinforcement being able to redistribute stress. An attempt was made to introduce a quasi-plastic character into the mechanisms transferring shear by appropriately shaping the shear reinforcement. Experimental material tests covered the determination of the strength and deformability of straight glass fiber-reinforced polymer (GFRP) bars and GFRP headed bars. Experimental studies of shear-reinforced beams with GFRP stirrups and GFRP headed bars were carried out. This allowed a direct comparison of the shear behavior of beams reinforced with standard GFRP stirrups and a new type of shear reinforcement: GFRP headed bars. Experimental studies demonstrated that GFRP headed bars could be used as shear reinforcement in concrete beams. Unlike GFRP stirrups, these bars allow stress redistribution in bars cut by a diagonal crack [16].

The paper by [17] examines the effect of PBO (P-phenylene benzobisoxazole)–FRCM (fabric-reinforced cementitious matrix) reinforcement on the stiffness of eccentrically compressed reinforced concrete columns. Reinforcement with FRCM consists of bonding composite meshes to the concrete substrate by means of mineral mortar. Longitudinal and/or transverse reinforcements made of PBO (P-phenylene benzobisoxazole) mesh were applied to the analyzed column specimens. When assessing the stiffness of the columns, the focus was on the effect of the composite reinforcement itself, the value and eccentricity of the longitudinal force, and the decrease in the modulus of elasticity of the concrete, with increasing stress intensity in the latter. Dependencies between the change in the elasticity modulus of the concrete and the change in the stiffness of the tested specimens were examined. The relevant standards, providing methods of calculating the stiffness of composite columns, were used in the analysis. Regarding columns, which were strengthened only transversely with PBO mesh, reinforcement increases their load capacity, and at the same time, the stiffness of the columns increases due to the confinement of the cross-section. The stiffness depends on the destruction of the concrete core inside its composite jacket. In the case of columns with transverse and longitudinal reinforcement, the presence of longitudinal reinforcement reduces longitudinal deformations. The columns failed at higher stiffness values in the whole range of the eccentricities [17].

The paper by [18] presents the results of an experimental investigation into stop-splayed scarf joints which was carried out as part of a research program at the Wroclaw University of Science and Technology. A brief description of the characteristics of scarf and splice joints appearing in historical buildings is provided, with special reference to stop-splayed scarf joints (so-called “bolt of lightning” joints) which were widely used, for example, in Italian Renaissance architecture. Analyses and studies of scarf and splice joints in bent elements as presented in the literature are reviewed, along with selected examples of analyses and research on tensile joints. It is worth noting that the authors in practically all the cited literature draw attention to the need for further research in this area. Next, the results of the authors’ own research on beams with stop-splayed scarf joints, strengthened using various methods, e.g., by means of drawbolts (metal screws), steel clamps and steel clamps with wooden pegs, which were subjected to four-point bending tests, are presented. Load-deflection plots were obtained for load-bearing to bending of each beam in relation to the load-bearing of a continuous reference beam. A comparative analysis of the results obtained for each beam series is presented, along with conclusions and directions for further research [18].

Non-destructive testing of concrete for defects detection, using acoustic techniques, is currently performed mainly by human inspection of recorded images [19]. The images consist of the inside of the examined elements, obtained from testing devices such as the ultrasonic tomograph. However, such an automatic inspection is time-consuming, expensive, and prone to errors. To address some of these problems, this paper aims to evaluate a convolutional neural network (CNN) toward an automated detection of flaws in concrete elements using ultrasonic tomography. There are two main stages in the proposed methodology. In the first stage, an image of the inside of the examined structure is obtained and recorded by performing ultrasonic tomography-based testing. In the second stage, a convolutional neural network model is used for the automatic detection of defects and flaws in the recorded image. In this work, a large and pre-trained CNN is used. It was fine-tuned on a small set of images collected during laboratory tests. Lastly, the prepared model was applied for detecting flaws. The obtained model has proven to be able to accurately detect defects in examined concrete elements. The presented approach for automatic detection of flaws is being developed with the potential to not only detect defects of one type but also to classify various types of defects in concrete elements [19].

The reliability and safety of power transmission depend first and foremost on the state of the power grid, and mainly on the state of the high-voltage power line towers [20]. The steel structures of existing power line supports (towers) have been in use for many years. Their in-service time, the variability in structural, thermal and environmental loads, the state of foundations (displacement and degradation), the corrosion of supporting structures and lack of technical documentation are essential factors that have an impact on the operating safety of the towers. The tower state assessment used to date, consisting of finding the deviation in the supporting structure apex, is insufficient because it omits the other necessary condition, the stress criterion, which is not to exceed allowable stress values. Moreover, in difficult terrain conditions, the measurement of the tower deviation is very troublesome, and for this reason, it is often not performed. This paper presents a stress-and-strain analysis of the legs of 110 kV power line truss towers with a height of 32 m. They have been in use for over 70 years and are located in especially difficult geotechnical conditions—one of them is in a gravel mine on an island surrounded by water, and the other stands on a steep, wet slope. Purpose-designed fiber Bragg grating (FBG) sensors were proposed for strain measurements. Real values of stresses arising in the tower legs were observed and determined over a period of one year. Validation was also carried out based on geodetic measurements of the tower apex deviation, and a residual magnetic field (RMF) analysis was performed to assess the occurrence of cracks and stress concentration zones [20].

The paper by [21] explores the microstructural evolution characteristics of tailings sand samples from different types of infiltration failure during the infiltration failure process. A homemade small infiltration deformation instrument is used to test the infiltration failure characteristics of the tailings sand during the infiltration failure process. Evolutionary characteristics of the internal microstructure pores and particle distribution were also studied. Using CT (computerized tomography) technology to establish digital image information, the distribution of the microscopic characteristics of the particle distribution and pore structure after tailing sand infiltration were studied. Microscopic analysis was also performed to analyze the microscopic process of infiltration and destruction, as well as to see the microscopic structural characteristics of the infiltration and destruction of the total tailings. The test results show that there are obvious differences in the microstructure characterization of fluid soil and piping-type infiltration failures. Microstructure parameters have a certain functional relationship with macro factors. Combining the relationship between macrophysical and mechanical parameters and microstructural parameters, new ideas for future research and the prevention of tailings sand infiltration and failure mechanisms are provided [21].

The paper by [22] presents the results of tests for flexural tensile strength (f_ct,fl_) and fracture energy (G_f_) in a three-point bending test of prismatic beams with notches, which were made from steel fiber-reinforced high-strength concrete (SFRHSC). The registration of the conventional force–displacement (F–δ) relationship and unconventional force-crack tip opening displacement (CTOD) relationship was made. On the basis of the obtained test results, estimations of the parameters f_ct,fl_ and G_f_ in the function of the fiber-reinforcement ratio were carried out. The obtained results were applied to building and validating a numerical model with the use of the finite element method (FEM). A non-linear concrete damaged plasticity model CDP was used for the description of the concrete. The obtained FEM results were compared with the experimental ones that were based on the assumed criteria. The usefulness of the flexural tensile strength and fracture energy parameters for defining the linear form of weakening of the SFRHSC material under tension was confirmed. The author’s own equations for estimating the flexural tensile strength and fracture energy of SFRHSC, as well as for approximating deflections (δ) of SFRHSC beams as the function of crack tip opening displacement (CTOD) instead of crack mouth opening displacement (CMOD), were proposed [22].

The accepted methods for testing concrete are not favorable for determining its heterogeneity [23]. The interpretation of the compressive strength result as a product of destructive force and cross-section area is burdened with significant understatements. It is assumed erroneously that this is the lowest value of strength at the height of the tested sample. The top layer of concrete floors often crumble, and the strength tested using sclerometric methods does not confirm the concrete class determined using control samples. That is why it is important to test the distribution of compressive strength in a cross-section of concrete industrial floors with special attention to surface top layers. This study presents strength tests of borehole material taken from industrial floors using the ultrasonic method, with exponential spot heads with a contact surface area of 0.8 mm^2^ and a frequency of 40 kHz. The presented research project anticipated the determination of strength for samples in various cross-sections at the height of elements and destructive strength in the strength testing machine. It was confirmed that for standard and big borehole samples, it is not possible to test the strength of concrete in the top layer of the floor by destructive methods. This can be achieved using the ultrasonic method. After the analysis, certain types of distributions of strength across concrete floor thickness were chosen from the completed research program. The gradient and anti-gradient of strength were proposed as new parameters for the evaluation of floor concrete quality [23].

Ventilated facades are becoming an increasingly popular solution for the external part of walls in buildings [24]. They may differ in many elements, among others, cladding (fiber cement boards, HPL plates, large-slab ceramic tiles, ACM panels, stone cladding), types of substructures, console supports, etc. The main element that characterizes ventilated facades is the use of an air cavity between the cladding and thermal insulation. Unfortunately, in some respects, they are not yet standardized and tested. Above all, the requirements for the falling-off of elements from ventilated facades during a fire are not precisely defined by, among other things, the lack of clearly specified requirements and testing. This is undoubtedly a major problem, as it significantly affects the safety of evacuation during a fire emergency. For the purposes of this article, experimental tests were carried out on a large-scale facade model, with two types of external facade cladding. The materials used as external cladding were fiber cement boards and large-slab ceramic tiles. The model of the large-scale test was 3.95 m × 3.95 m; the burning gas released from the burner was used as the source of fire. The facade model was equipped with thermocouples. The test lasted one hour, and the cladding materials showed different behavior during the test. Large-slab ceramic tiles seemed to be a safer form of external cladding for ventilated facades. Unfortunately, they were destroyed much faster, by about 6 min. Large-slab ceramic tiles were destroyed within the first dozen or so minutes, then their destruction did not proceed or was minimal. In the case of fiber cement boards, the destruction started from the eleventh minute and increased until the end of the test. The author referred the results of the large-scale test to testing on samples carried out by other authors. The results presented the convergence of the large-scale test with samples. External claddings were equipped with additional mechanical protection. The use of additional mechanical protection to maintain external cladding elements increases their safety but does not completely eliminate the problem of the falling-off of parts of the facade. As research on fiber cement boards and large-slab ceramic tiles has suggested, these claddings were a major hazard due to fall-off from the facade [24].

The aim of this study [25] was to investigate the effect of plasterboards’ humidity absorption on their performance. The specimens’ hydration procedure consisted of consecutive immersing in water and subsequent drying at room temperature. Such a procedure was performed to increase the moisture content within the material volume. The microstructural observations of five different plasterboard types were performed through optical and scanning electron microscopy. The deterioration of their properties was evaluated using a three-point bending test and a subsequent ultrasonic (ultrasound testing (UT)) longitudinal wave velocity measurement. Depending on the material porosity, a loss of UT wave velocity from 6% to 35% and a considerable decrease in material strength from 70% to 80% were observed. Four types of approximated formulae were proposed to describe the dependence of UT wave velocity on the board moisture content. It was found that the proposed UT method could be successfully used for the on-site monitoring of plasterboards’ hydration processes [25].

Concrete structure joints are filled in mainly in the course of sealing works ensuring protection against the influence of water. This paper by [26] presents the methodology for testing the mechanical properties of ESD pseudoplastic resins (E—elastic deformation, S—strengthening control, D—deflection control) recommended for concrete structure joint fillers. The existing standards and papers concerning quasi-brittle cement composites do not provide an adequate point of reference for the tested resins. The lack of a standardized testing method hampers the development of materials universally used in expansion joint fillers in reinforced concrete structures, as well as the assessment of their properties and durability. An assessment of the obtained results by referring to the reference sample has been suggested in the article. A test stand and a method of assessing the mechanical properties results (including adhesion to the concrete surface) of pseudoplastic resins in the axial tensile test have been presented [26].

The compaction index is one of the most important technological parameters during asphalt pavement construction, which may be negatively affected by the wrong asphalt paving machine setting, weather conditions, or the mix temperature. Presented in [27], this laboratory study analyzes the asphalt mix properties in case of inappropriate compaction. The reference mix was designed for an AC 11 S wearing layer (asphalt concrete for a wearing layer with maximum grading of 11 mm). Asphalt mix samples used in the tests were prepared using a Marshall device with the compaction energy of 2 × 20, 2 × 35, 2 × 50, and 2 × 75 blows, as well as in a roller compactor where the slabs were compacted to various heights: 69.3 mm (+10% of nominal height), 66.2 mm (+5%), 63 mm (nominal), and 59.9 mm (−5%), which resulted in different compaction indexes. Afterward, the samples were cored from the slabs. Both Marshall samples and cores were tested for air void content, stiffness modulus in three temperatures, indirect tensile strength, and resistance to water and frost indicated by the ITSR value. It was found that either an insufficient or excessive level of compaction can cause a negative effect on the road surface performance [27].

The importance of surface roughness and its non-destructive examination has often been emphasized in structural rehabilitation. The innovative procedure presented in [28] enables the estimation of concrete-to-concrete strength, based on a combination of low-cost, area-limited tests and geostatistical methods. The new method removes the shortcomings of the existing one, i.e., it is neither qualitative nor subjective. The interface strength factors, cohesion and friction, can be estimated accurately based on the collected data on surface texture. The data acquisition needed to create digital models of the concrete surface can be performed by terrestrial close-range photogrammetry or other methods. In the presented procedure, limitations to the availability of concrete surfaces are overcome by the generation of subsequential Gaussian random fields (via height profiles) based on the semivariograms fitted to the digital surface models. In this way, the randomness of the surface texture is reproduced. The selected roughness parameters, such as mean valley depth and, most importantly, the geostatistical semivariogram parameter sill, were transformed into contact bond strength parameters based on the available strength tests. The proposed procedure estimates the interface bond strength based on the geostatistical methods applied to the numerical surface model and can be used in practical and theoretical applications [28].

The core part of a hybrid truss bridge is the connection joint that combines the concrete chord and steel truss-web members [29]. To study the mechanical behavior and failure mode of steel–concrete connection joints in a hybrid truss bridge, static model tests were carried out on two connection joints at the scale of 1:3, under a horizontal load that was provided by a loading jack mounted on the vertical reaction wall. The specimen design, experimental setup and testing procedure were introduced. In the experiment, the displacement, strain level, concrete crack and experimental phenomena were factually recorded. Compared with the previous study results, the experimental results in this study demonstrated that the connection joints had an excellent bearing capacity and deformability. The minimum ultimate load and displacement of the two connection joints were 5200 kN and 59.01 mm, respectively. Moreover, the connection joints exhibited multiple failure modes, including the fracture of gusset plates, the slippage of high-strength bolts, the local buckling of compressive splice plates, the fracture of tensile splice plates and concrete cracking. Additionally, the strain distribution of the steel–concrete connection joints followed certain rules. It is expected that the findings from this paper may provide a reference for the design and construction of steel–concrete connection joints in hybrid truss bridges [29].

The static elastic modulus (*Ec*) and compressive strength (*fc*) are critical properties of concrete [30]. When determining *Ec* and *fc*, concrete cores are collected and subjected to destructive tests. However, destructive tests require certain test permissions and large sample sizes. Hence, it is preferable to predict *Ec* using the dynamic elastic modulus (*Ed*), through non-destructive evaluations. A resonance frequency test performed according to ASTM C215-14, and a pressure wave (P-wave) measurement conducted according to ASTM C597M-16, are typically used to determine *Ed*. Recently, developments in transducers have enabled the measurement of shear wave (S-wave) velocities in concrete. Although various equations have been proposed for estimating *Ec* and *fc* from *Ed*, their results deviate from experimental values. Thus, it is necessary to obtain a reliable *Ed* value for accurately predicting *Ec* and *fc*. In this study, *Ed* values were experimentally obtained from P-wave and S-wave velocities in the longitudinal and transverse modes; *Ec* and *fc* values were predicted using these *Ed* values through four machine learning (ML) methods: support vector machine, artificial neural networks, ensembles, and linear regression. Using ML, the prediction accuracy of *Ec* and *fc* was improved by 2.5–5% and 7–9%, respectively, compared with the accuracy obtained using classical or normal-regression equations. By combining ML methods, the accuracy of the predicted *Ec* and *fc* was improved by 0.5% and 1.5%, respectively, compared with the optimal single variable results [30].

The paper by [31] describes tests conducted to identify the mechanisms occurring during the fracture of single-edge notches loaded in three-point bending (SENB) specimens made from an Al–Ti laminate. The experimental tests were complemented with microstructural analyses of the specimens’ fracture surfaces and an in-depth analysis of acoustic emission (AE) signals. The paper presents the application of the AE method to identify fracture processes in the layered Al–Ti composite, using a non-hierarchical method for clustering AE signals (k-means) and analyses using waveform time domain, fast Fourier transform (FFT Real) and waveform continuous wavelet, based on the Morlet wavelet. These analyses made it possible to identify different fracture mechanisms in Al–Ti composites, which is very significant for the assessment of the safety of structures made from this material [31].

The aspects regarding the stiffness of the connections between the beams that support the storage pallets and the uprights are very important in the analysis of the displacements and stresses in the storage racking systems. The main purpose of the paper by [32] is to study the effects of both upright thickness and tab connector types on rotational stiffness and on the capable bending moment of the connection. For this purpose, 18 different groups of beam-connector-upright assemblies are prepared by combining three types of beams (different sizes of the box cross-section), three kinds of upright profiles (with a different thickness of the section walls), and two types of connectors (four-tab connectors and five-tab connectors). Flexural tests were carried out on 101 assemblies. For the assemblies containing the uprights with a thickness of 1.5 mm, the five-tab connector leads to a higher value of the capable moment and higher rotational stiffness than similar assemblies with four-tab connectors. A contrary phenomenon happens in the case of the assemblies containing those upright profiles having a thickness of 2.0 mm, regarding the capable design moment. It is shown how the safety coefficient of connection depends on both the rotational stiffness and capable bending moment [32].

Concrete shrinkage is a phenomenon that results in a decrease in volume in the composite material during the curing period. The method for determining the effects of restrained shrinkage is described in Standard ASTM C 1581/C 1581M–09a. This article [33] shows the calibration of measuring rings with respect to the theory of elasticity, and the analysis of the relationship of steel ring deformation to high-performance concrete tensile stress as a function of time. Steel rings equipped with strain gauges are used for the measurement of strain during the compression of the samples. The strain is caused by the shrinkage of the concrete ring specimen that tightens around steel rings. The method allows registering the changes to the shrinkage process over time and evaluating the susceptibility of concrete to cracking. However, the standard does not focus on the details of the mechanical design of the test bench. To acquire accurate measurements, the test bench needs to be calibrated. Measurement errors may be caused by an improper, uneven installation of strain gauges, imprecise geometry of the steel measuring rings, or incorrect equipment settings. The calibration method makes it possible to determine the stress in a concrete sample, leading to its cracking at the specific deformation of the steel ring [33].

The article by [34] proposes using the acoustic emission (AE) method to evaluate the degree of change in the mechanical parameters of fiber cement boards. The research was undertaken after a literature review, due to the lack of a methodology that would allow nondestructive assessment of the strength of cement–fiber elements. The tests covered the components cut out from a popular type of board available on the construction market. The samples were subjected to environmental (soaking in water, cyclic freezing–thawing) and exceptional (burning with fire and exposure to high temperature) factors, and then to three-point bending strength tests. The adopted conditions correspond to the actual working environment of the boards. When applying the external load, AE signals were generated which were then grouped into classes, and initially assigned to specific processes occurring in the material. The frequencies occurring over time for the tested samples were also analyzed, and microscopic observations were made to confirm the suppositions based on the first part of the tests. Comparing the results obtained from a group of samples subjected to environmental and exceptional actions, significant differences were noted between them, which included the types of recorded signal class, the frequency of events, and the construction of the microstructure. The degradation of the structure, associated with damage to the fibers or their complete destruction, results in the generation under load of AE signals that indicate the uncontrolled development of scratches, and a decrease in the frequency of these events. According to the authors, the methodology used allows the control of cement–fiber boards in use. The registration and analysis of active processes under the effect of payloads makes it possible to distinguish mechanisms occurring inside the structure of the elements, and to formulate a quick response to the situation when the signals indicate a decrease in the strength of the boards [34].

The article by [35] discusses one of the methods of dielectric constant determination in a continuous way, which is the determination of its value based on the amplitude of the wave reflected from the surface. Based on tests performed on model asphalt slabs, the research presented how the value of the dielectric constant changes, depending on the atmospheric conditions of the measured surface (dry, covered with water film, covered with ice, covered with snow, covered with de-icing salt). Coefficients correcting dielectric constants of hot mix asphalt (HMA) determined in various surface atmospheric conditions were introduced. It was proposed to determine the atmospheric conditions of the pavement with the use of wavelet analysis in order to choose the proper dielectric constant correction coefficient and, therefore, improve the accuracy of the pavement layer thickness estimation based on the ground-penetrating radar (GPR) method [35].

Phenomena occurring during the curing of concrete can decrease its mechanical properties, specifically its strength and serviceability, even before it is placed [36]. This is due to excessive stresses caused by temperature gradients, moisture changes, and chemical processes arising during the concreting and in hardened concrete. At stress concentration sites, microcracks form in the interfacial transition zones (ITZ) in the early phase and propagate deeper into the cement paste or to the surface of the element. Microcracks can contribute to the development of larger cracks, reduce the durability of structures, limit their serviceability, and, in rare cases, lead to their failure. It is thus important to search for a tool that allows objective assessment of damage initiation and development in concrete. The objectivity of the assessment lies in it being independent of the constituents and additives used in the concrete or of external influences. The acoustic emission-based method presented in this paper allows damage detection and identification in the early age of concrete (before loading) for different concrete compositions, curing conditions, temperature variations, and in reinforced concrete. As such, this method is an objective and effective tool for damage process detection [36].

The authors of [37] suggest a wire-mesh method to classify the particle shape of large amounts of aggregate. This method is controlled by the tilting angle and opening size of the wire mesh. The more rounded the aggregate particles, the more they roll on the tilted wire mesh. Three different sizes of aggregate, 11–15, 17–32, and 33–51 mm, were used for assessing their roundness after classification, using the sphericity index to sort them into rounded, sub-rounded/sub-angular, and angular. The aggregate particles with different sphericities were colored differently and then used for classification via the wire-mesh method. The opening sizes of the wire mesh were 6, 11, and 17 mm, and its frame was 0.5 m wide and 1.8 m long. The ratio of aggregate size to mesh-opening size was between 0.6 and 8.5. The wire mesh was inclined at various angles of 10°, 15°, 20°, 25°, and 30° to evaluate the rolling degree of the aggregates. The aggregates were rolled and remained on the wire mesh between 0.0–0.6, 0.6–1.2, and 1.2–1.8 m, depending on their sphericity. A tilting angle of 25° was the most suitable angle for classifying aggregate size ranging from 11–15 mm, while the most suitable angle for aggregate sizes of 17–32 and 33–51 mm was 20°. The best ratio for the average aggregate size to mesh-opening size for the aggregate roundness classification was 2 [37].

Taking into account the possibilities offered by two imaging methods, X-ray microcomputed tomography (µCT) and two-dimensional optical scanning, this article by [38] discusses the possibility of using these methods to assess the internal structure of spun concrete, particularly its composition after hardening (Michałek 2020). To demonstrate the performance of the approach based on imaging, laboratory techniques based on physical and chemical methods were used as verification. A comparison of the obtained results of applied research methods was carried out on samples of spun concrete, characterized by the layered structure of the annular cross-section. Samples were taken from the power pole E10.5/6c (Strunobet-Migacz, Lewin Brzeski, Poland) made by one of the Polish manufacturers of prestressed concrete E-poles precast in steel molds. The validation shows that optical scanning followed by appropriate image analysis is an effective method for evaluation of the spun concrete internal structure. In addition, such analysis can significantly complement the results of the laboratory methods used so far. In a fairly simple way, through the porosity image, it can reveal improperly selected parameters of concrete spinning, such as speed and time, and, through the distribution of cement content in the cross-section of the element, it can indicate compliance with the requirement for the corrosion durability of spun concrete. The research methodology presented in the paper can be used to improve the production process of poles made of spun concrete; it can be an effective tool for verifying concrete structure [38].

In the study by [39], the effects of the mixing conditions of waste-paper sludge ash (WPSA) on the strength and bearing capacity of controlled low-strength material (CLSM) were evaluated, and the optimal mixing conditions were used to evaluate the strength characteristics of CLSM with recyclable WPSA. The strength and bearing capacity of CLSM with WPSA were evaluated using unconfined compressive strength tests and plate bearing tests, respectively. The unconfined compressive strength test results show that the optimal mixing conditions for securing 0.8–1.2 MPa of target strength under 5% of cement content conditions can be obtained when both WPSA and fly ash are used. This is because WPSA and fly ash, which act as binders, have a significant impact on overall strength when the cement content is low. The bearing capacity of weathered soil increased from 550 to 575 kPa over time, and CLSM with WPSA increased significantly, from 560 to 730 kPa. This means that the bearing capacity of CLSM with WPSA was 2.0% higher than that of weathered soil immediately after construction; furthermore, it was 27% higher at 60 days of age. In addition, the allowable bearing capacity of CLSM corresponding to the optimal mixing conditions was evaluated, and it was found that this value increased by 30.4% until 60 days of age. This increase rate was 6.7 times larger than that of weathered soil (4.5%). Therefore, based on the allowable bearing capacity calculation results, CLSM with WPSA was applied as a sewage pipe backfill material. It was found that CLSM with WPSA performed better as backfill and was more stable than soil immediately after construction. The results of this study confirm that CLSM with WPSA can be utilized as sewage-pipe backfill material [39].

Non-destructive testing (NDT) methods are an important means to detect and assess rock damage [40]. To better understand the accuracy of NDT methods for measuring damage in sandstone, this study compared three NDT methods, including ultrasonic testing, electrical impedance spectroscopy (EIS) testing, computed tomography (CT) scan testing, and a destructive test method, elastic modulus testing. Sandstone specimens were subjected to different levels of damage through cyclic loading, and different damage variables derived from five different measured parameters—longitudinal wave (P-wave) velocity, first-wave amplitude attenuation, resistivity, effective bearing area and the elastic modulus—were compared. The results show that the NDT methods all reflect the damage levels for sandstone accurately. The damage variable derived from the P-wave velocity is more consistent with the other damage variables, and the amplitude attenuation is more sensitive to damage. The damage variable derived from the effective bearing area is smaller than that derived from the other NDT measurement parameters. Resistivity provides a more stable measure of damage, and damage derived from the acoustic parameters is less stable. By developing P-wave velocity-to-resistivity models based on theoretical and empirical relationships, it was found that differences between these two damage parameters can be explained by differences between the mechanisms through which they respond to porosity, since the resistivity reflects pore structure, while the P-wave velocity reflects the extent of the continuous medium within the sandstone [40].

The H spin-lattice relaxometry (T_1_, longitudinal) of cement pastes with 0 to 0.18 wt % polycarboxylate superplasticizers (PCEs) at intervals of 0.06 wt % from 10 min to 1210 min was investigated in [41]. Results showed that the main peak in T_1_ relaxometry of cement pastes was shorter and lower along with the hydration times. PCEs delayed and lowered this main peak in the T_1_ relaxometry of cement pastes at 10 min, 605 min and 1210 min, which was highly correlated to its dosages. In contrast, PCEs increased the total signal intensity of T_1_ of cement pastes at these three times, which still correlated to its dosages. Both changes of the main peak in T_1_ relaxometry and the total signal intensity of T_1_ revealed interferences in evaporable water during cement hydration by the dispersion mechanisms of PCEs. The time-dependent evolution of the weighted average T_1_ of cement pastes with different PCEs between 10 min and 1210 min was found to be regular to the four-stage hydration mechanism of tricalcium silicate [41].

Control of technical parameters obtained by ready-mixed concrete may be carried out at different stages of the development of concrete properties, and by different participants involved in the construction investment process [42]. According to the European Standard EN 206 “Concrete—specification, performance, production and conformity”, mandatory control of concrete conformity is conducted by the producer during production. As shown by the subject literature, statistical criteria set out in the standard, including the method for concrete quality assessment based on the concept of concrete family, continue to evoke discussions and raise doubts. This justifies seeking alternative methods for concrete quality assessment. This paper presents a novel approach to quality control and the classification of concrete based on combining statistical and fuzzy theories as a means of representation of two types of uncertainty: random uncertainty and information uncertainty. In concrete production, a typical situation when fuzzy uncertainty can be taken into consideration is the conformity control of concrete compressive strength, which is conducted to confirm the declared concrete class. The proposed procedure for quality assessment of a concrete batch is based on defining the membership function for the considered concrete classes and establishing the degree of belonging to the considered concrete class. It was found that concrete classification set out by the standard includes too many concrete classes of overlapping probability density distributions, and the proposed solution was to limit the scope of compressive strength to every second class so as to ensure the efficacy of conformity assessment conducted for concrete classes and concrete families. The proposed procedures can lead to two types of decisions: non-fuzzy (crisp) or fuzzy, which point to possible solutions and their corresponding preferences. The suggested procedure for quality assessment allows researchers to classify a concrete batch in a fuzzy way with the degree of certainty less than or equal to 1. The results obtained confirm the possibility of employing the proposed method for quality assessment in the production process of ready-mixed concrete [42].

In recent years, the application of fiber-reinforced plastics (FRPs) as structural members has been promoted [43]. Metallic bolts and rivets are often used for the connection of FRP structures, but there are some problems caused by corrosion and stress concentration at the bearing position. Fiber-reinforced thermoplastics (FRTPs) have attracted attention in composite material fields because they can be remolded by heating and manufactured at excellent speed compared with thermosetting plastics. In this paper, we propose and evaluate the connection method using rivets produced from FRTPs for FRP members. It was confirmed through material tests that an FRTP rivet provides stable tensile, shear, and bending strength. Then, it was clarified that a non-clearance connection could be achieved by the proposed connection method, so initial sliding was not observed, and connection strength linearly increased as the number of FRTP rivets increased through the double-lapped tensile shear tests. Furthermore, the joint strength of the beam using FRTP rivets could be calculated with high accuracy, using the method for bolt joints in steel structures through a four-point beam bending test [43].

Polymer pipes are used in the construction of underground gas, water, and sewage networks [44]. During exploitation, various external forces work on the pipeline which cause its deformation. In this paper, numerical analysis and experimental investigations of polyethylene pipe deformation at different external load values (500, 1000, 1500, and 2000 N) were performed. The authors measured the strains of the lower and upper surfaces of the pipe during its loading moment using resistance strain gauges, which were located on the pipe at equal intervals. The results obtained from computer simulation and experimental studies were comparable. An innovative element of the research presented in the article is the recognition of the impact of the proposed values of the load of polyethylene pipe on the change in its deformation [44].

The shear and particle-crushing characteristics of the failure plane (or shear surface) in catastrophic mass movements are examined with a ring shear apparatus, which is generally employed owing to its suitability for large deformations [45]. Based on the results of previous experiments on waste materials from abandoned mine deposits, we employed a simple numerical model based on ring shear testing using the particle flow code (PFC^2D^). We examined drainage, normal stress, and the shear velocity-dependent shear characteristics of landslide materials. For shear velocities of 0.1 and 100 mm/s and normal stress (NS) of 25 kPa, the numerical results are in good agreement with those obtained from experimental results. The difference between the experimental and numerical results of the residual shear stress was approximately 0.4 kPa, for NS equal to 25 kPa and 0.9 kPa for NS equal to 100 kPa, for both drained and undrained conditions. In addition, we examined the particle-crushing effect during shearing using the frictional work concept in PFC. We calculated the work done by friction at both peak and residual shear stresses, and then used the results as crushing criteria in the numerical analysis. The frictional work at peak and the residual shear stresses ranged from 303 kPa·s to 2579 kPa·s for the given drainage and normal stress conditions. These results showed that clump particles were partially crushed at peak shear stress [45].

The main objective of the research presented in [46] was to develop a solution to the global problem of using steel waste obtained during rubber recovery during tire recycling. A detailed comparative analysis of the mechanical and physical features of concrete composite with the addition of recycled steel fibers (RSF) in relation to the steel fiber concrete commonly used for industrial floors was conducted. A study was carried out using micro-computed tomography and the scanning electron microscope to determine the fibers’ characteristics, including the EDS spectrum. In order to designate full performance of the physical and mechanical features of the novel composite, a wide range of tests was performed, with particular emphasis on the determination of the tensile strength of the composite. This parameter, appointed by tensile strength testing for splitting, residual tensile strength testing (3-point test), and a wedge splitting test (WST), demonstrated the increase of tensile strength (vs. unmodified concrete) by 43%, 30%, and 70% respectively to the method. The indication of the reinforced composite’s fracture characteristics using the digital image correlation (DIC) method allowed the researchers to illustrate the map of deformation of the samples during WST. The novel composite was tested in reference to the circular economy concept and showed 31.3% lower energy consumption and 30.8% lower CO_2_ emissions than a commonly used fiber concrete [46].

Existing buildings, especially historical buildings, require periodic or situational diagnostic tests [47]. If a building is in use, advanced non-destructive or semi-destructive methods should be used. In the diagnosis of reinforced concrete structures, tests allowing assessment of the condition of the reinforcement and concrete cover are particularly important. The article presents the non-destructive and semi-destructive research methods that are used for such tests, as well as the results of tests performed for selected elements of a historic water tower structure. The assessment of the corrosion risk of the reinforcement was carried out with the use of a semi-destructive galvanostatic pulse method. The protective properties of the concrete cover were checked by the carbonation test and the phase analysis of the concrete, for which X-ray diffractometry and thermal analysis methods were used. In order to determine the position of the reinforcement and to estimate the concrete cover thickness distribution, a ferromagnetic detection system was used. The comprehensive application of several test methods allowed mutual verification of the results and the drawing of reliable conclusions. The results indicated a very poor state of reinforcement, loss in the depth of cover, and sulfate corrosion [47].

The paper by [48] presents the possibility of using low-module polypropylene dispersed reinforcement (E = 4.9 GPa) to influence the load-deflection correlation of cement composites. Problems have been indicated regarding the improvement of elastic range when using that type of fiber as compared with a composite without reinforcement. It was demonstrated that it was possible to increase the ability to carry stress in the Hooke’s law proportionality range in the mortar and paste types of composites reinforced with low-module fibers, i.e., V_f_ = 3% (in contrast to concrete composites). The possibility of having good strengthening and deflection control in order to limit the catastrophic destruction process was confirmed. This paper identifies the problem of deformation assessment in composites with significant deformation capacity. Determining the effects of reinforcement based on a comparison with a composite without fibers is suggested as a reasonable approach, as it enables the comparison of results obtained by various universities under different research conditions [48].

Arcan shear tests with digital image correlation were used to evaluate the shear modulus and shear stress–strain diagrams in the plane defined by two principal axes of the material orthotropy [49]. Two different orientations of the grain direction, as compared to the direction of the shear force in specimens, were considered: perpendicular and parallel shear. Two different ways were used to obtain the elastic properties based on the digital image correlation (DIC) results from the full-field measurement and from the virtual strain gauges with the linear strains: perpendicular to each other and directed at an angle of π/4 to the shearing load. In addition, their own continuum structural model for the failure analysis in the experimental tests was used. The constitutive relationships of the model were established in the framework of the mathematical multi-surface elastoplasticity for the plane stress state. The numerical simulations performed by the finite element program after implementation of the model demonstrated the failure mechanisms from the experimental tests [49].

After obtaining the value of shear wave velocity (*V_S_*) from the bender elements test (BET), the shear modulus of soils at small strains (*G_max_*) can be estimated [50]. Shear wave velocity is an important parameter in the design of geo-structures subjected to static and dynamic loading. While bender elements are increasingly used in both academic and commercial laboratory test systems, there remains a lack of agreement when interpreting the shear wave travel time from these tests. Based on the test data of 12 Warsaw glacial quartz samples of sand, two different approaches were primarily examined for determining *V_S_*. They are both related to the observation of the source and received *BE* signal, namely, the first time of arrival and the peak-to-peak method. These methods were performed through visual analysis of BET data by the authors, so that subjective travel time estimates were produced. Subsequently, automated analysis methods from the GDS Bender Element Analysis Tool (BEAT) were applied. Here, three techniques in the time-domain (TD) were selected, namely, the peak-to-peak, the zero-crossing, and the cross-correlation functions. Additionally, a cross-power spectrum calculation of the signals was completed, viewed as a frequency-domain (FD) method. Final comparisons between subjective observational analyses and automated interpretations of BET results showed good agreement. There is compatibility, especially between the two methods of the first time of arrival and cross-correlation, which the authors considered the best interpreting techniques for their soils. Moreover, the laboratory tests were performed on compact, medium, and well-grained sand samples with different curvature coefficients and mean grain sizes [50].

The reduction in natural resources and aspects of environmental protection necessitate alternative uses for waste materials in the area of construction [51]. Recycling is also observed in road construction, where mineral–cement emulsion (MCE) mixtures are applied. The MCE mix is a conglomerate that can be used to make the base layer in road pavement structures. MCE mixes contain reclaimed asphalt from old, degraded road surfaces, aggregate for improving the gradation, asphalt emulsion, and cement as a binder. The use of these ingredients, especially cement, can cause shrinkage and cracks in road layers. The article presents selected issues related to the problem of cracking in MCE mixtures. The authors of the study focused on reducing the cracking phenomenon in MCE mixes by using an innovative cement binder with recycled materials. The innovative cement binder, based on dusty by-products from cement plants, also contributes to the optimization of the recycling process in road surfaces. The research was carried out in the fields of stiffness, fatigue life, crack resistance, and shrinkage analysis of mineral–cement emulsion mixes. It was found that it was possible to reduce the stiffness and cracking in MCE mixes. The use of innovative binders will positively affect the durability of road pavements [51].

The windblown sand-induced degradation of glass panels influences the serviceability and safety of these panels. In this study, the degradation of glass panels subject to windblown sand at different impact velocities and impact angles was studied based on a sandblasting test simulating a sandstorm [52]. After the glass panels were degraded by windblown sand, the surface morphology of the damaged glass panels was observed using scanning electron microscopy, and three damage modes were found: a cutting mode, smash mode, and plastic deformation mode. The mass loss, visible light transmittance, and effective area ratio values of the glass samples were then measured to evaluate the effects of the windblown sand on the panels. The results indicate that, at high abrasive feed rates, the relative mass loss of the glass samples decreases initially and then remains steady with increases in impact time, whereas it increases first and then decreases with an increase in impact angle, such as that for ductile materials. Both the visible light transmittance and effective area ratio decrease with increases in the impact time and velocities. There exists a positive linear relationship between the visible light transmittance and effective area ratio [52].

Durability tests against fungal action for wood-plastic composites are carried out in accordance with European standard ENV 12038, but the authors of the manuscript try to prove that the assessment of the results performed according to these methods is imprecise and suffers from a significant error [53]. Fungal exposure is always accompanied by high humidity, so the result of tests made by such a method is always burdened with the influence of moisture, which can lead to a wrong assessment of the negative effects of the action of the fungus itself. The paper (Wiejak 2021) has shown a modification of such a method that separates the destructive effect of fungi from moisture accompanying the test’s destructive effect. The functional properties selected to prove the proposed modification are changes in the mass and bending strength after subsequent environmental exposure. It was found that the intensive action of moisture measured in the culture chamber at about (70 ± 5)%, i.e., for 16 weeks, at (22 ± 2) °C, which was the fungi culture in the accompanying period, led to changes in the mass of the wood-plastic composites, amounting to 50% of the final result of the fungi resistance test, and changes in the bending strength amounting to 30–46% of the final test result. As a result of this research, the correction for assessing the durability of wood–polymer composites against biological corrosion has been proposed. The laboratory tests were compared with the products’ test results following three years of exposure to the natural environment [53].

Reduced maintenance costs of concrete structures can be ensured by efficient and comprehensive condition assessment [54]. Ground-penetrating radar (GPR) has been widely used in the condition assessment of reinforced concrete structures and it provides completely non-destructive results in real-time. It is mainly used for locating reinforcement and determining concrete cover thickness. More recently, research has focused on the possibility of using GPR for reinforcement corrosion assessment. In this paper, an overview of the application of GPR in the corrosion assessment of concrete is presented. A literature search and study selection methodology were used to identify the relevant studies. First, the laboratory studies are shown. After that, the studies for application on real structures are presented. The results have shown that the laboratory studies have not fully illuminated the influence of the corrosion process on the GPR signal. In addition, no clear relationship was reported between the results of the laboratory studies and the on-site inspection. Although GPR has a long history in the condition assessment of structures, it needs more laboratory investigations to clarify the influence of the corrosion process on the GPR signal [54].

The paper by [55] attempts to compare three methods of testing floor slip resistance and the resulting classifications. Polished, flamed, brushed, and grained granite slabs were tested. The acceptance angle values (α_ob_) obtained through the shod ramp test, slip resistance value (SRV), and sliding friction coefficient (μ) were compared in terms of the correlation between the series, the precision of each method, and the classification results assigned to each of the three obtained indices. It was found that the evaluation of a product for slip resistance was strongly related to the test method used and the resulting classification method. This influence was particularly pronounced for low-roughness slabs. This would result in risks associated with inadequate assessments that could affect the safe use of buildings and facilities [55].

Standard sensors for the measurement and monitoring of temperature in civil structures are liable to mechanical damage and electromagnetic interference [56]. A system of purpose-designed fiber-optic FBG sensors offers a more suitable and reliable solution—the sensors can be directly integrated with the load-bearing structure during construction, and it is possible to create a network of fiber-optic sensors to ensure not only temperature measurements but also measurements of strain and of the moisture content in the building envelope. The paper describes the results of temperature measurements of a building’s two-layer wall using optical fiber Bragg grating (FBG) sensors, and of a three-layer wall using equivalent classical temperature sensors. The testing results can be transmitted remotely. In the first stage, the sensors were tested in a climatic test chamber to determine their characteristics. The paper describes the test results of temperature measurements carried out in the winter season for two multilayer external walls of a building, in relation to the environmental conditions recorded at that time, i.e., outdoor temperature, relative humidity, and wind speed. Cases are considered with the biggest difference in the level of the relative humidity of air recorded in the observation period. It was found that there is greater convergence between the theoretical and the real temperature distribution in the wall for high levels (~84%) of the outdoor air’s relative humidity, whereas, at the humidity level of ~49%, the difference between theoretical and real temperature histories is substantial and totals up to 20%. A correction factor is proposed for the theoretical temperature distribution [56].

The paper by [57] deals with a complex analysis of acoustic emission signals that were recorded during freeze-thaw cycles in test specimens produced from air-entrained concrete. An assessment of the resistance of concrete to the effects of freezing and thawing was conducted on the basis of signal analysis. Since the experiment simulated the testing of concrete in a structure, a concrete block with a height of 2.4 m and width of 1.8 m was produced to represent a real structure. When the age of the concrete was two months, samples were obtained from the block by core drilling and were subsequently used to produce test specimens. Testing of the freeze-thaw resistance of concrete employed both destructive and non-destructive methods including the measurement of acoustic emission, which took place directly during the freeze-thaw cycles. The recorded acoustic emission signals were then meticulously analyzed. The aim of the conducted experiments was to verify whether measurement using the acoustic emission method during freeze-thaw (F-T) cycles is more sensitive to the degree of damage of concrete than the more commonly employed construction testing methods. The results clearly demonstrate that the acoustic emission method can reveal changes (e.g., minor cracks) in the internal structure of concrete, unlike other commonly used methods. The analysis of the acoustic emission signals using a fast Fourier transform revealed a significant shift of the dominant frequency toward lower values when the concrete was subjected to freeze-thaw cycling [57].

An original experimental method was used to investigate the influence of water and road salt with an anti-caking agent on the material used in pavement construction layers [58]. This method allowed the monitoring of material changes resulting from the influence of water and road salt with an anti-caking agent over time. The experiment used five different mineral road mixes, which were soaked separately in water and brine for two time intervals (2 days and 21 days). Then, each sample of the mix was subjected to tests of the complex module using the four-point bending (4PB-PR) method. The increase in mass of the soaked samples and the change in value of the stiffness modulus were analyzed. Exemplary tomographic (X-ray) imaging was performed to confirm the reaction of the road salt and anti-caking agent (lead agent) with the material. Based on the measurements of the stiffness modulus and absorption, the correlations of the mass change and the value of the stiffness modulus were determined, which may be useful in estimating the sensitivity of mixes to the use of winter maintenance agents, e.g., road salt with an anti-caking agent (sodium chloride). It was found that the greatest changes occur with mixes intended for base course layers (mineral cement mix with foamed asphalt (MCAS) and mineral-cement-emulsion mixes (MCE)), and that the smallest changes occur for mixes containing highly modified asphalt (HIMA) [58].

Cracking in non-load-bearing internal partition walls is a serious problem that frequently occurs in new buildings in the short term after putting them into service, or even before completion of construction [59]. Sometimes, it is so considerable that it cannot be accepted by the occupiers. The article presents tests of cracking in ceramic walls with a door opening connected in a rigid and flexible way along vertical edges. The first analyses were conducted using the finite element method (FEM), and afterward, the measurements of deformations and stresses in walls on deflecting floors were performed at full scale in the actual building structure. The measurements enabled the authors to determine floor deformations leading to the cracking of walls and to establish a dependency between the values of tensile stresses within the area of the door opening corners and their location along the length of walls, and the type of vertical connection with the structure [59].

The paper by [60] discusses the problems connected with the long-term exploitation of reinforced concrete post-tensioned girders. The scale of problems in the world related to the number of cable post-tensioned concrete girders built in the 1950s and still in operation is very large and possibly has very serious consequences. The paper presents an analysis and evaluation of the results of measurements of the deflection and strength and homogeneity of concrete in cable–concrete roof girders of selected industrial halls located in Poland, exploited for over 50 years. On the basis of the results of displacement monitoring in the years 2009 to 2020, the maximum increments of deflection of the analyzed girders were determined. Non-destructive, destructive, and indirect evaluation methods were used to determine the compressive strength of concrete. Within the framework of the indirect method recommended in standard PN-EN 13791, a procedure was proposed by the authors to modify the so-called base curve for determining compressive strength. Due to the age of the analyzed structural elements, a correction factor for the age of the concrete was taken into account in the strength assessment. The typical value of the characteristic compressive strength was within the range of 20.3–28.4 MPa. As a result of the conducted tests, the concrete class assumed in the design was not confirmed, and its classification depended on the applied test method. The analyzed girders, despite their long-term exploitation, can still be used for years, on the condition that regular periodical inspections of their technical condition are carried out. The authors emphasize the necessity for a permanent and cyclic diagnostic process and monitoring of the geometry of girders, as they are expected to operate much longer than was assumed by their designers [60].

The paper by [61] presents an implementation of purpose-designed optical fiber Bragg grating (FBG) sensors intended for the monitoring of real values of strain in reinforced road structures in areas of mining activity. Two field test stations are described. The first enables analysis of the geogrid on concrete and ground subgrades. The second models the situation of subsoil deformation due to mining activity at different external loads. The paper presents a system of optical fiber sensors registering strain and temperature dedicated to the investigated concrete mattress. Laboratory tests were performed to determine the strain characteristic of the FBG sensor–geogrid system with respect to standard load. As a result, it was possible to establish the dependence of the geogrid strain on the forces occurring within it. This may be the basis for an analysis of the mining activity effect on right-of-way structures during precise strain measurements of a geogrid using FBG sensors embedded within it. The analysis of the results of measurements in the aspect of forecasted and actual static and dynamic effects of mining on the stability of a reinforced road structure is of key importance for detailed management of road investment, and for the appropriate repair and modernization management of the road structure [61].

Geopolymer concrete (GPC) offers a potential solution for sustainable construction by utilizing waste materials [62]. However, the production and testing procedures for GPC are quite cumbersome and expensive, which can slow down the development of mix design and the implementation of GPC. The basic characteristics of GPC depend on numerous factors such as the type of precursor material, type of alkali activators and their concentration, and liquid to solid (precursor material) ratio. To optimize time and cost, artificial neural networks (ANN) can be a lucrative technique for exploring and predicting GPC characteristics. In this study, the compressive strength of fly-ash-based GPC, with bottom ash as a replacement for fine aggregates, as well as fly ash, is predicted using a machine learning-based ANN model. The data inputs are taken from the literature as well as in-house lab scale testing of GPC. The specifications of GPC specimens act as input features of the ANN model to predict compressive strength as the output, while minimizing error. Fourteen ANN models are designed which differ in the backpropagation training algorithm, the number of hidden layers, and the neurons in each layer. The performance analysis and comparison of these models in terms of mean squared error (MSE) and coefficient of correlation (R) resulted in a Bayesian regularized ANN (BRANN) model for the effective prediction of compressive strength of fly-ash and bottom-ash based geopolymer concrete [62].

The paper by [63] analyzes the issue of reduction of load capacity in fiber cement board during a fire. Fiber cement boards were put under the influence of fire by using a large-scale facade model. Such a model is a reliable source of knowledge regarding the behavior of facade cladding and the way fire spreads. One technical solution for external walls—a ventilated facade—is gaining popularity and is used more and more often. However, the problem of the destruction during a fire of a range of different materials used in external facade cladding is insufficiently recognized. For this study, the authors used fiber cement boards as the facade cladding. Fiber cement boards are fiber-reinforced composite materials, mainly used for facade cladding, but are also used as roof cladding, drywall, drywall ceilings and floorboards. This paper analyzes the effect of fire temperatures on facade cladding using a large-scale facade model. Samples were taken from external facade cladding materials that were mounted on the model at specific locations above the combustion chamber. Subsequently, three-point bending flexural tests were performed, and the effects of temperature and the integrals of temperature and time functions on the samples were evaluated. The three-point bending flexural test was chosen because it is a universal method for assessing fiber cement boards, as cited in Standard EN 12467. The test also allows easy reference to results in other literature [63].

The paper by [64] presents the possibilities of determining the range of stresses preceding the critical destruction process in cement composites, with the use of micro-events identified by means of a sound spectrum. The presented test results refer to the earlier papers in which micro-events (destruction processes) were identified, but without determining the stress level of their occurrence. This paper indicates a correlation of the stress level corresponding to the elastic range with the occurrence of micro-events in traditional and quasi-brittle composites. Tests were carried out on beams (with and without reinforcement) subjected to four-point bending. In summary, it is suggested that the conclusions can be extended to other test cases (e.g., compression strength), which should be confirmed by the appropriate tests. The paper also indicates a need for further research to identify micro-events. The correct recognition of micro-events is important for the safety and durability of traditional and quasi-brittle cement composites [64].

The paper by [65] contains the results of a newly developed residual-state creep test, performed to determine the behavior of a selected geomaterial in the context of reactivated landslides. Soil and rock creep is a time-dependent phenomenon in which deformation occurs under constant stress. Based on the examination results, it was found that the tested clayey material (from Kobe, Japan) shows tertiary creep behavior only under shear stress higher than the residual strength condition, and primary and secondary creep behavior under shear stress lower than or equal to the residual strength condition. Based on the data, a model for predicting the critical or failure time is introduced. The time until the occurrence of the conditions necessary for unlimited creep on the surface is estimated. As long-term precipitation and infiltrating water in the area of the landslides are identified as the key phenomena initiating collapse, the work focuses on the prediction of landslides, with identified surfaces of potential damage as a result of changes in the saturation state. The procedure outlined is applied to a case study, and considerations as to when the necessary safety work should be carried out are presented [65].

The article by [66] is focused on the medium-term negative effect of groundwater on the underground grout elements. This is the physical–mechanical effect of groundwater, which is known as erosion. We conducted a laboratory verification of the erosional resistance of grout mixtures. A new test apparatus was designed and developed, since there is no standardized method for testing at present. An erosion stability test of grout mixtures and the technical solutions of the apparatus for the test’s implementation are described. This apparatus was subsequently used for the experimental evaluation of the erosional stability of silicate grout mixtures. Grout mixtures with activated and non-activated bentonite are tested. The stabilizing effect of cellulose relative to erosion stability has also been investigated. The specimens of grout mixtures were exposed to flowing water stress for a set period of time. The erosional stabilities of the grout mixtures were assessed on the basis of weight loss (WL) as a percentage of initial specimen weight. The lower the grout mixture weight loss, the higher its erosional stability, and vice versa [66].

## 3. Conclusions

As mentioned at the beginning, this issue was proposed and organized as a means of presenting recent developments in the field of non-destructive testing of materials in civil engineering. For this reason, the articles highlighted in this issue relate to different aspects of the testing of different materials in civil engineering, from building materials and elements to building structures. Interesting results, with significance for the materials, were obtained, and all of the papers have been precisely described.
